# Antennas for Licensed Shared Access in 5G Communications with LTE Mid- and High-Band Coverage

**DOI:** 10.3390/s23042095

**Published:** 2023-02-13

**Authors:** Khaled M. Morshed, Debabrata K. Karmokar, Karu P. Esselle

**Affiliations:** 1 School of Engineering, Macquarie University, Sydney, NSW 2109, Australia; 2 UniSA STEM, University of South Australia (UniSA), Mawson Lakes, SA 5095, Australia; 3 School of Electrical and Data Engineering, University of Technology Sydney, Ultimo, NSW 2007, Australia

**Keywords:** licensed shared access (LSA), 5G, long-term evolution (LTE), dual band, wideband, hand-held device antenna

## Abstract

Two novel antennas are presented for mobile devices to enable them to access both licensed shared access (LSA) bands (1452–1492 and 2300–2400 MHz) and all the long-term evolution (LTE) mid (1427–2690 MHz) and high (3400–3800 MHz) bands, together with the GSM1800, GSM1900, UMTS, and 3.3 GHz WiMAX bands. These antennas do not require any passive or active lumped elements for input impedance matching. One of them is a dual-band antenna and the other is a wideband antenna. Both antennas have high efficiency in all the LSA bands, as well as the mid- and high-LTE bands, and nearly omnidirectional radiation patterns in the mid band. In the high band, the radiation patterns of the wideband antenna are less directional than those of the dual-band antenna. The wideband antenna was fabricated and tested and the measurements demonstrated that it had good wideband performance in a wide frequency range from 1.37 to 4 GHz, covering all the above-mentioned bands.

## 1. Introduction

The rapidly growing demands of mobile traffic can be supported by fifth-generation (5G) wireless communication systems [1,2,3,4,5,6]. An application of cognitive radio technology named licensed shared access (LSA) has been proposed for 5G mobile communication systems to allow the sharing of spectrum among mobile network operators [7,8,9,10]. In Europe, a 1.5 GHz band (1452–1492 MHz) and a 2.3 GHz band (2300–2400 MHz) have been allocated for LSA service. The proposed LSA service has been tested in the 2.3 GHz band in Europe and more bands are expected to be allocated in the future [8,11,12].

Antennas with very large bandwidths are required in mobile devices to cover many bands, including, for example, the long-term evolution (LTE) mid (1427–2690 MHz) band, LTE high (3400–3800 MHz) band, DCS/GSM1800, and PCS/GSM1900 bands. The LTE mid and high bands consist of 16 frequency-division duplexing (FDD) bands (LTE 1–4, 7, 9–11, 15, 16, 21–25, 30) and 11 time-division duplexing (TDD) bands (LTE 33-43) [13]. Several antennas have been proposed with multiband or wideband operation for wireless wide-area network (WWAN)/LTE hand-held devices to cover the frequency range from 1710 to 2690 MHz [14,15,16,17,18]. The antenna presented in [19] covers a wide frequency band from 1585 to 2195 MHz. However, this antenna covers only a part of the LTE mid band, and the performance of the antenna has not been verified after integrating the antenna into the substrate of a mobile phone. On the other hand, metal-framed antennas are reported in [20,21]. The antenna in [21] covers only the LTE mid band, whereas the antenna in [20] covers both the LTE mid and high bands. Some antennas have been reported to cover both the 1710–2690 MHz (LTE mid) and 3400–3800 MHz (LTE high) frequency ranges [22,23,24,25,26]. Recently, an antenna was presented in [27] that covers both frequency bands. However, in order to achieve acceptable impedance matching ($|S_{11}|<-6$ dB) in the above-mentioned bands, an extra lumped-element-based circuit is required with the feed section. The reported antennas cover 13 FDD and all TDD LTE bands. For example, the maximum antenna coverage reported in [17,24,28] is 1630–2740 and 3300–3800 MHz. Though the antenna presented in [28] is compact and covers the 1555–2690 and 3100–3700 MHz frequency bands, three nulls have been observed in the high-band radiation patterns. Moreover, it does not cover the entire high band. Mobile hand-held antennas that can cover the following FDD–LTE bands are in high demand: LTE11 (1427–1501 MHz), LTE21 (1447–1511 MHz), and LTE24 (1525–1661 MHz). The LTE11 and LTE21 bands have been deployed in Japan by ‘au mobile’ and ‘NTT Docomo’, respectively, and the LTE24 band in the USA by ‘Ligado Networks’ [29,30].

Therefore, mobile devices require antennas to cover the 0.69–0.96, 1.42–2.69, and 3.3–3.8 GHz frequency bands without any nulls in the radiation patterns in all bands. Moreover, these antennas should be compact enough to fit in a mobile device while allowing enough space for other electronic circuits and the battery. For presentation simplicity, the above three bands are referred to as the low, mid, and high bands, respectively, throughout this paper. Several reported antennas have sufficiently wide operating bandwidths to cover the low band [14,15,16,17,18,22,23,24,25]. Considering the requirements of current and forthcoming mobile communication systems, we aimed to design antennas to cover the mid and high bands while maintaining an antenna area and ground clearance similar to the antennas reported in [14,15,16,17,18,22,23,24,25].

In this paper, first, a planar antenna is presented for hand-held devices to cover the LSA frequency bands. Later, two novel antenna designs are described. One is a dual-band antenna with a planar structure loaded with an I-shaped vertical plate, and the other is a wideband antenna with a planar structure loaded with an L-shaped vertical plate. Both antennas cover all the LSA bands, as well as the LTE mid and high bands. These antennas occupy a small area and they do not require any passive or active lumped components for input impedance matching. In this paper, we describe the design and working principle of all three antennas. The wideband antenna, which is more compact than the dual-band antenna and planar antenna, was prototyped and tested experimentally.

This paper is organized as follows. First, the design and working principle of the planar antenna (Ant#1) are described in Section 2. Second, the compact dual-band antenna (Ant#2) is presented in Section 3. Third, a wideband antenna (Ant#3), which is even more compact than the previous two antennas, is described in Section 4. Ant#3 is fabricated and the measured results are discussed in Section 5. A comparison of all three antennas is also given in this section. Finally, Section 6 concludes the paper.

## 2. A Planar Antenna for LSA Bands

This section presents a planar antenna (Ant#1) for the 1.5 and 2.3 GHz LSA bands.

### 2.1. Antenna Configuration

**Figure 1:**
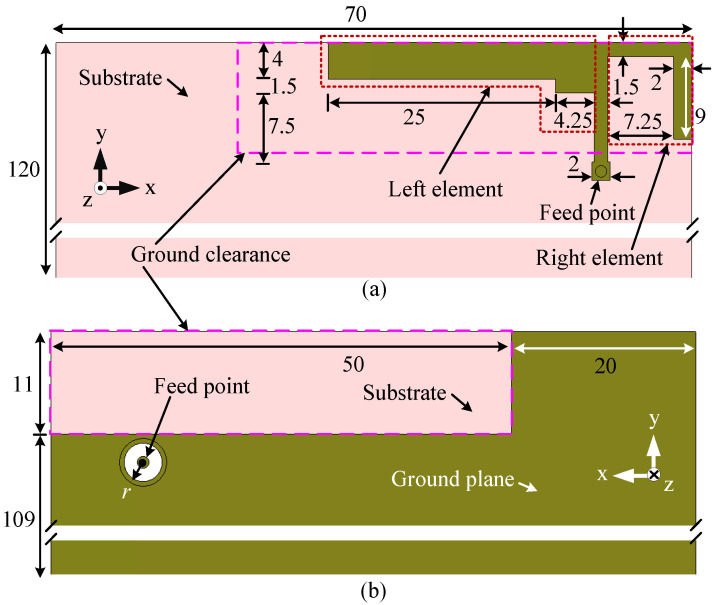
Configuration of the planar antenna (Ant#1) for LSA operation (1452–1492 and 2300–2400 MHz): (**a**) top view and (**b**) bottom view. All dimensions are in millimeters.

This printed antenna (Ant#1) is designed to operate in the 1452–1492 and 2300–2400 MHz LSA bands and its detailed geometry with top and bottom views is shown in Figure 1. The total area of the antenna is 40 mm × 15 mm, or 0.18$\lambda_{0}$ × 0.07$\lambda_{0}$, where $\lambda_{0}$ is the free-space wavelength at 1.38 GHz. For the simulation, the antenna is assumed to be fed by an SMA connector at the bottom. To ensure isolation between the ground plane and the center pin of the SMA connector, a circular portion of copper with radius *r* is etched off from the ground plane, as shown in Figure 1b. The antenna is placed in the top-right corner of a mobile device substrate with dimensions of 120 mm × 70 mm. A substrate commonly used in mobile devices is FR-4, with a relative permittivity ($\epsilon_{r}$) and loss tangent (tanδ) of 4.3 and 0.025, respectively. Its thickness is assumed to be 0.8 mm. The overall area of the ground plane is equal to the area of the substrate. A segment of copper with dimensions of 50 mm × 11 mm is removed from the top-right corner of the ground plane, as shown in Figure 1b.

The proposed antenna is composed of two elements: an L-shaped element to the left of the feed point (left element) to cover the 1.5 GHz LSA band and an L-shaped element to the right (right element) to cover the 2.3 GHz LSA band. CST Microwave Studio is used for the design and analyses of the antenna. The dimensions are optimized using a parameter analysis conducted in CST for good impedance matching.

### 2.2. Design Procedure and Working Principle

**Figure 2:**
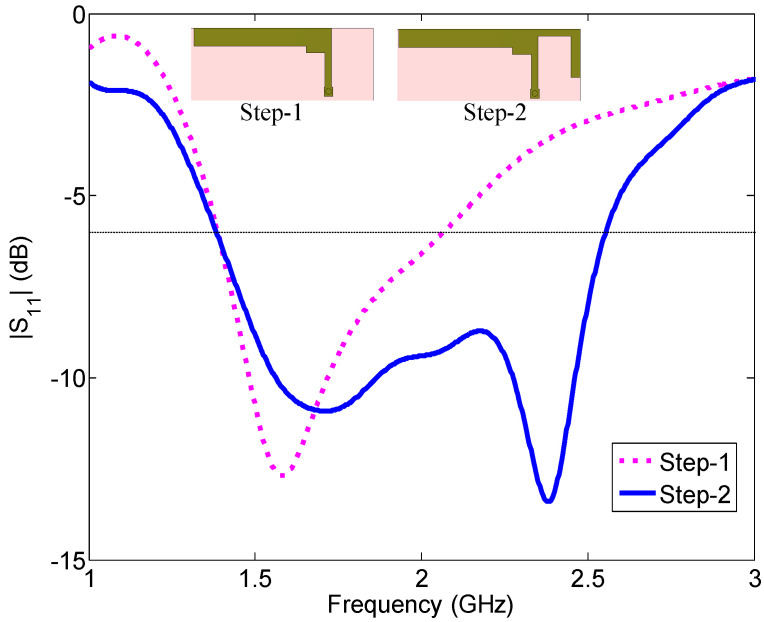
Two steps of the evolution of the planar antenna (Ant#1) to cover the two LSA bands.

The design of this LSA antenna starts with a simple L-shaped antenna, which is shown as Step 1 in Figure 2. This antenna has a −6 dB reflection coefficient bandwidth of 670 MHz (1.39–2.06 GHz), as shown in Figure 2, and resonates at 1.58 GHz. The effective length of the antenna (Step-1) is 44 mm.

In the next step, our target is to achieve an operating band that could cover the higher LSA band (2.3 GHz band), together with the lower LSA band (1.5 GHz band). In order to have another resonance at the higher LSA band, an L-shaped right element is added to the antenna element in Step 1, and the reflection coefficient curve is shown in Figure 2. The second antenna (Step 2) has a wide −6 dB reflection coefficient bandwidth of 1170 MHz (1.38–2.55 GHz) and covers both LSA bands, together with 12 FDD and 7 TDD LTE bands in the LTE mid band. The antenna resonates at 1.71 and 2.38 GHz within the 1.5 and 2.3 GHz LSA bands, respectively. The effective length of the right element is 31 mm, which is equal to a quarter wavelength at 2.38 GHz, and that of the left element is 44 mm, which is equal to a quarter wavelength at 1.71 GHz. Note that the electrical lengths are calculated with respect to the free-space wavelength.

**Figure 3:**
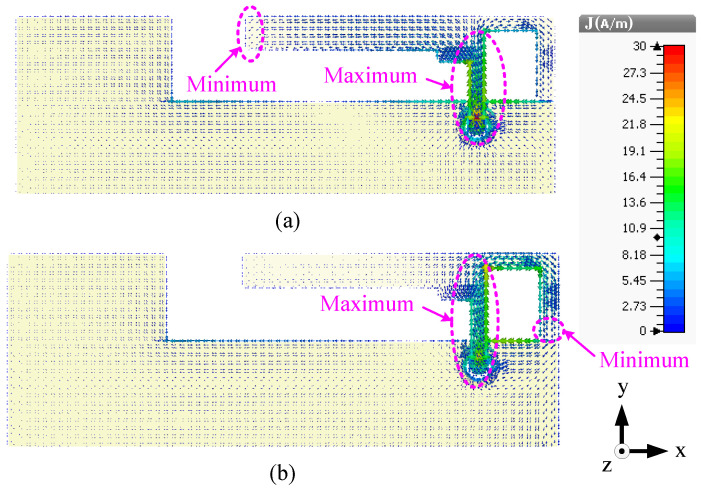
Surface current distributions of the planar antenna (Ant#1) operating in the LSA bands at (**a**) 1.8 and (**b**) 2.6 GHz.

Now let us study the surface current distribution, as shown in Figure 3, at 1.8 and 2.6 GHz in order to further understand the operation of the antenna. At both frequencies, the surface current is at a maximum around the feed point and a minimum at the end of the left or right element, meaning that the antenna resonates in the quarter-wavelength modes at both frequencies. The left element has strong currents at 1.8 GHz and the right element has strong currents at 2.6 GHz. However, in both cases, moderate currents are observed in the ground plane close to the antenna structure.

This planar antenna covers both the LSA bands but only a part of the mid-LTE band. In the next section, a dual-band antenna is presented to cover the entire LTE mid and high bands, together with the two LSA bands.

### 2.3. Radiation Performance

**Figure 4:**
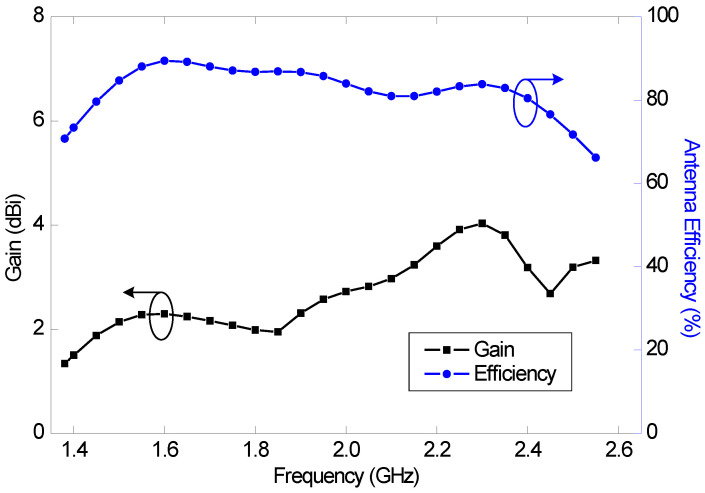
Predicted antenna efficiency and gain of the planar LSA antenna (Ant#1).

The antenna gain variation within the reflection coefficient bandwidth is 2.3 dB and the minimum and maximum antenna gains are 1.3 and 3.6 dBi, respectively. The minimum and maximum efficiencies are 66% and 89%, respectively. The efficiency and gain variation within the reflection coefficient bandwidth are shown in Figure 4.

**Figure 5:**
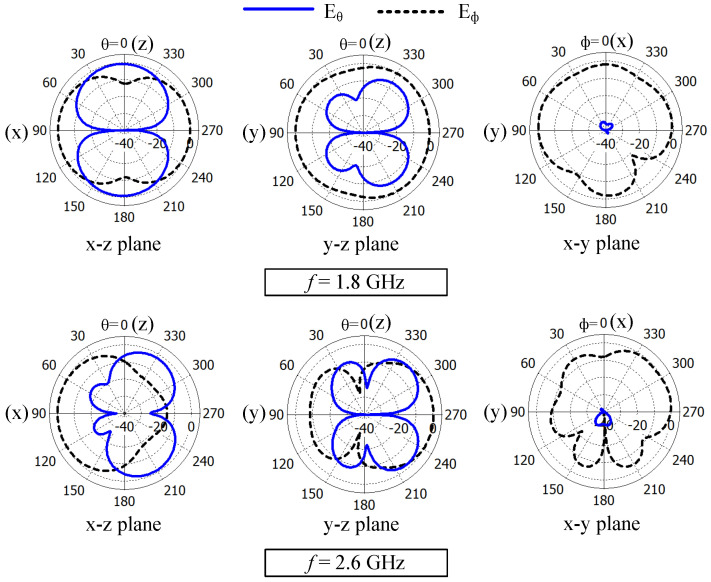
Normalized radiation patterns of the planar LSA antenna (Ant#1) at 1.8 and 2.6 GHz.

Figure 5 shows the normalized $E_{\phi }$ and $E_{\theta }$ radiation patterns of Ant#1 in the three principal planes at 1.8 and 2.6 GHz. Here, the patterns are normalized with respect to the maximum of the corresponding principal plane. From the presented results, no nulls can be observed at 1.8 GHz but a null can be observed at 2.6 GHz in the x–y plane radiation patterns. The $E_{\phi }$ radiation in the y–z plane at 1.8 GHz is more uniform (i.e., somewhat omnidirectional) compared to the $E_{\phi }$ radiation in the same plane at 2.6 GHz. An almost directed $E_{\phi }$ radiation pattern can be observed in the x–z plane at 2.6 GHz.

## 3. Dual-Band Antenna for LSA Bands and LTE Mid and High Bands

In this section, the design and performance of a dual-band antenna (Ant#2) are described.

### 3.1. Antenna Configuration

**Figure 6:**
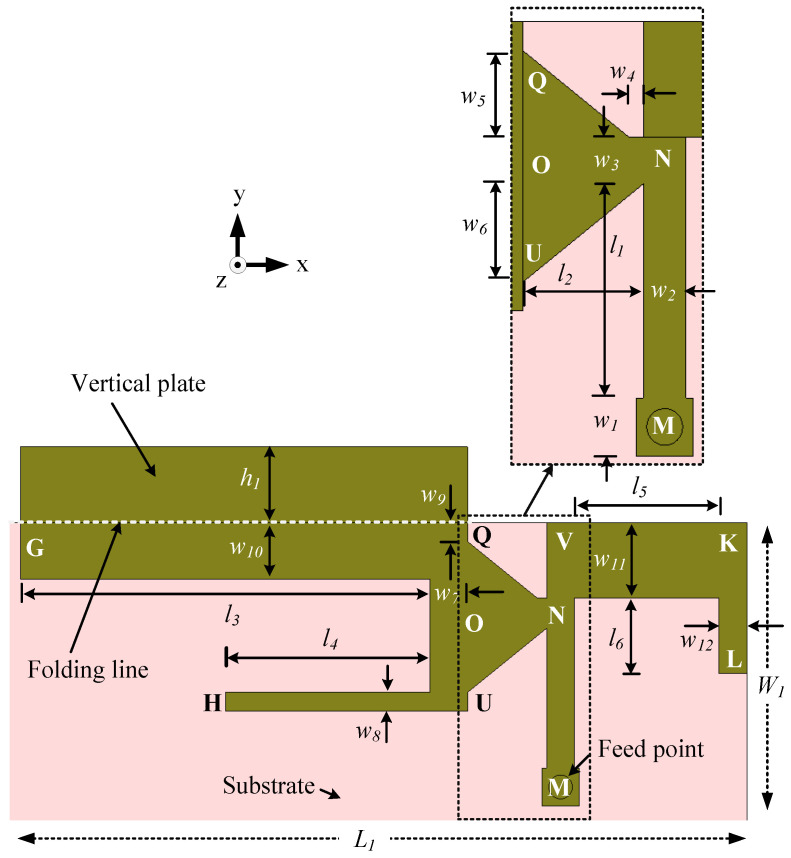
Configuration of the dual-band antenna (Ant#2) for the mid (1427–2690 MHZ) and high (3300–3800 MHz) bands. For better presentation, the vertical plate is unfolded along the +y-axis.

The dual-band antenna is designed to operate in the 1427–2690 and 3300–3800 MHz bands to support LSA, TDD/FDD LTE, UMTS, DCS, PCS, GSM1800, GSM1900, WLAN (2.4 GHz), and WiMAX (2.5 and 3.3 GHz), as shown in Figure 6. There is a small vertical plate in the antenna, which is shown as unfolded along the +y-axis in Figure 6 for better presentation. The substrate size, positioning of the antenna, feed arrangement, ground clearance, and back view are similar to Ant#1, as shown in Figure 1. The overall dimensions of the antenna are 39 mm × 15 mm × 4 mm or 0.18$\lambda_{1}$× 0.08$\lambda_{1}$× 0.02$\lambda_{1}$, where $\lambda_{1}$ is the free-space wavelength at 1.39 GHz (the lowest frequency of the antenna’s first operating band, i.e., the mid band). Although the antenna has a small vertical plate, the area occupied by this antenna is 3% less than that occupied by Ant#1.

This antenna is composed of three elements: an L-shaped element MNO in the middle, a U-shaped large element GQUH loaded with the vertical plate on the left, and an L-shaped element VKL on the right. Here, the vertical plate is aligned with the edge GQ. The dimensions are optimized using a parameter analysis conducted in CST for good impedance matching. The optimized design parameters of the dual-band antenna (Ant#2) are listed in Table 1.

### 3.2. Design Procedure and Working Principle

**Figure 7:**
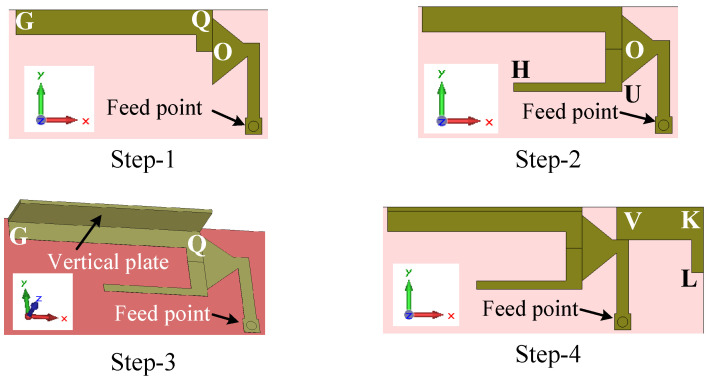
The four steps of the evolution of the dual-band antenna (Ant#2).

**Figure 8:**
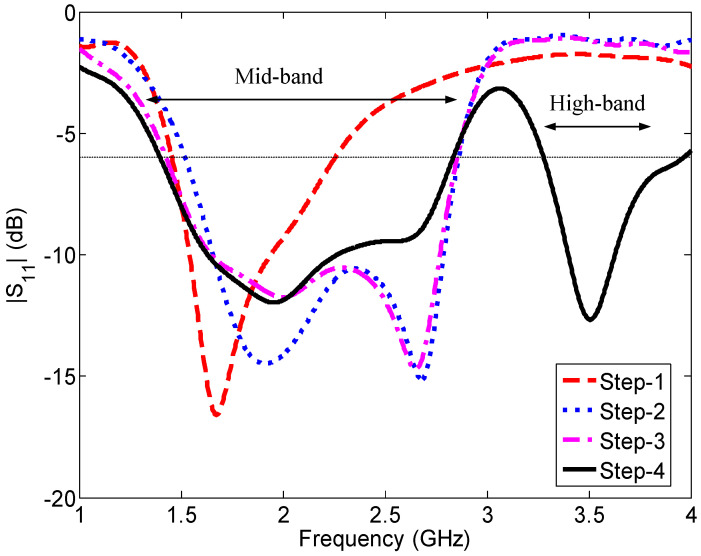
Reflection coefficient for each step in the dual-band antenna (Ant#2) evolution.

The design procedure of Ant#2 is divided into two main steps. The first step is to design an antenna for the 1427–2690 MHZ band. The second step is to add a new element to cover the high (3300–3800 MHz) band. These two steps are divided into four basic steps that illustrate the working principle. The evolution of the antenna and the corresponding predicted reflection coefficients are shown in Figure 7 and Figure 8, respectively.

In Step 1, the middle element MNO (inset of Figure 6) is designed and optimized. The effective length of this element is $l_{ILE}=l_{1}+l_{2}+\left(w_{1}+w_{2}+w_{3}\right)/2=14.2$ mm. Step 1 is completed by adding an L-shaped strip OQG with an effective length of $w_{3}/2+w_{5}+w_{7}/2+l_{3}=26.8$ mm to the edge OQ so that the antenna covers the mid (1427–2690 MHz) band. The antenna then resonates at 1.67 GHz, as shown in Figure 8 (Step 1 curve). The effective length of the antenna ($l_{ILE}+26.8=41$ mm) is close to a quarter wavelength at 1.67 GHz.

In Step 2, an L-shaped strip OUH with a length of $w_{3}/2+w_{6}+w_{8}/2+l_{4}=15.7$ mm is attached to the edge OU of the feed element, extending the length of the antenna by 29.9 mm. As can be seen in Figure 8 (Step 2 curve), the antenna then resonates at two frequencies, 1.98 and 2.68 GHz, because of the two different electrical paths MNOQG and MNOUH, respectively. In this step, the −6 dB reflection coefficient bandwidth of the antenna in the lower band is 1.35 GHz (1.51–2.86 GHz).

However, the lower-frequency limit needed to be shifted to 1.42 GHz to cover all the LTE mid and LSA bands. To achieve this, a vertical plate of height $h_{1}$ is loaded onto the edge GQ in Step 3. The vertical plate shifts the lower frequency limit to 1.41 GHz, with a negligible variation in the upper-frequency limit (2.85 GHz).

Our next target is to cover the high band (3300–3800 MHz), together with the mid band, with the same antenna. To achieve this, an L-shaped element VKL with an effective length of $w_{2}+l_{5}+w_{12}+l_{6}$ is introduced into the top of the middle element MN in Step 4. The effective length (right side of the middle element) of the new element is 24.75 mm; it generated a resonance at 3.51 GHz.

The final antenna design (Figure 6) consists of three electrical paths MNOQG (=41 mm), MNUHOG (=56.7 mm), and MNVKL (=24.75 mm), which make the antenna resonate at 1.96, 2.65, and 3.51 GHz, respectively. At 1.96 and 3.51 GHz, the antenna resonates in the quarter-wavelength modes, and at 2.65 GHz, the antenna resonates in the half-wavelength mode. The −6 dB reflection coefficient bandwidths of this dual-band antenna are 1.45 GHz (1.39–2.84 GHz) and 700 MHz (3.27–3.97 GHz), respectively, covering the targeted mid (1427–2690 MHz) and high (3300–3800 MHz) bands.

**Figure 9:**
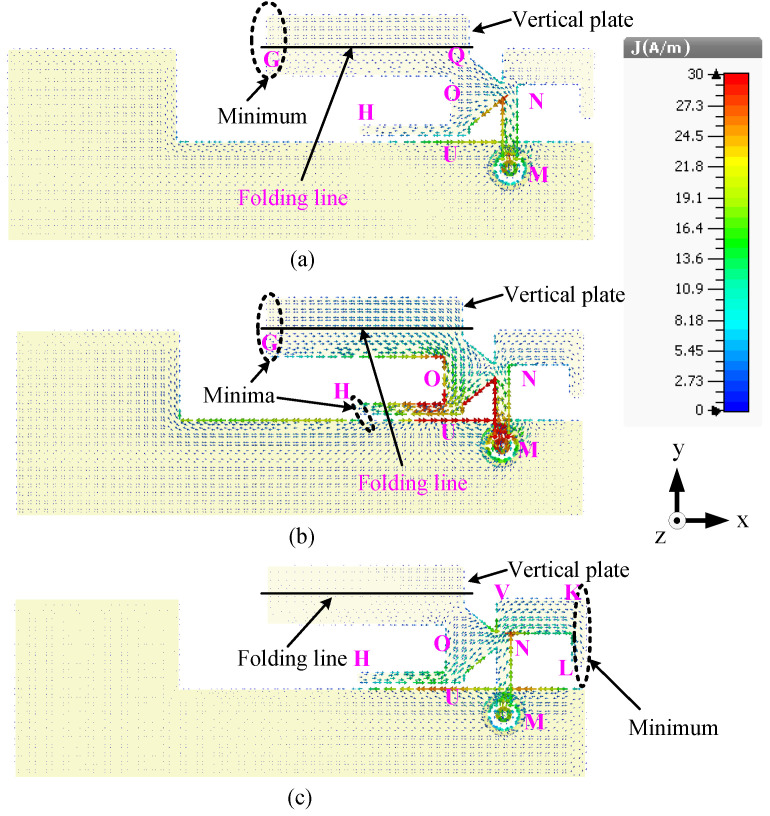
Surface current distributions of the dual-band antenna (Ant#2) at (**a**) 1.8, (**b**) 2.6, and (**c**) 3.6 GHz.

The current distributions of this antenna at the three frequencies are shown in Figure 9 to illustrate the working modes. For better presentation, the vertical plate is unfolded along the +y-axis. It can be seen in Figure 9a that the current is at a minimum close to G and a maximum close to M, meaning that the antenna resonates in the quarter-wavelength mode at 1.8 GHz. In addition, strong currents can be observed in the arm OUH at 1.8 GHz, meaning that the arm OUH is radiating at this frequency. At 2.6 GHz, the current is at a maximum close to M and there are two minima at H and G, as shown in Figure 9b, which also indicates that the antenna is resonating in the half-wavelength mode. Figure 9c illustrates that the antenna resonates in the quarter-wavelength mode at 3.6 GHz since the current is at a maximum at M and a minimum at edge KL.

### 3.3. Radiation Performance

**Figure 10:**
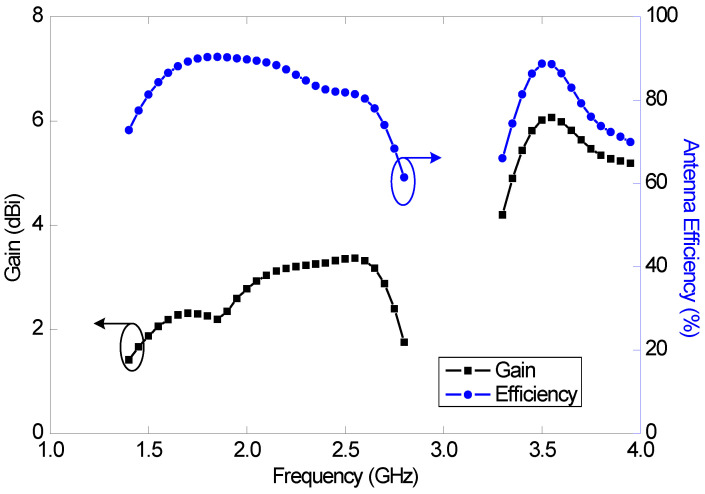
Predicted antenna efficiency and realized gain of the dual-band antenna (Ant#2).

The predicted antenna efficiency and gain as a function of frequency are shown in Figure 10. The maximum antenna efficiencies in the mid and high bands are 90% and 88%, respectively, and the minimum antenna efficiencies are 62% and 66%, respectively. The realized gain varies between 1.4 and 3.4 dBi in the mid band, and between 4.2 and 6.1 dBi in the high band.

**Figure 11:**
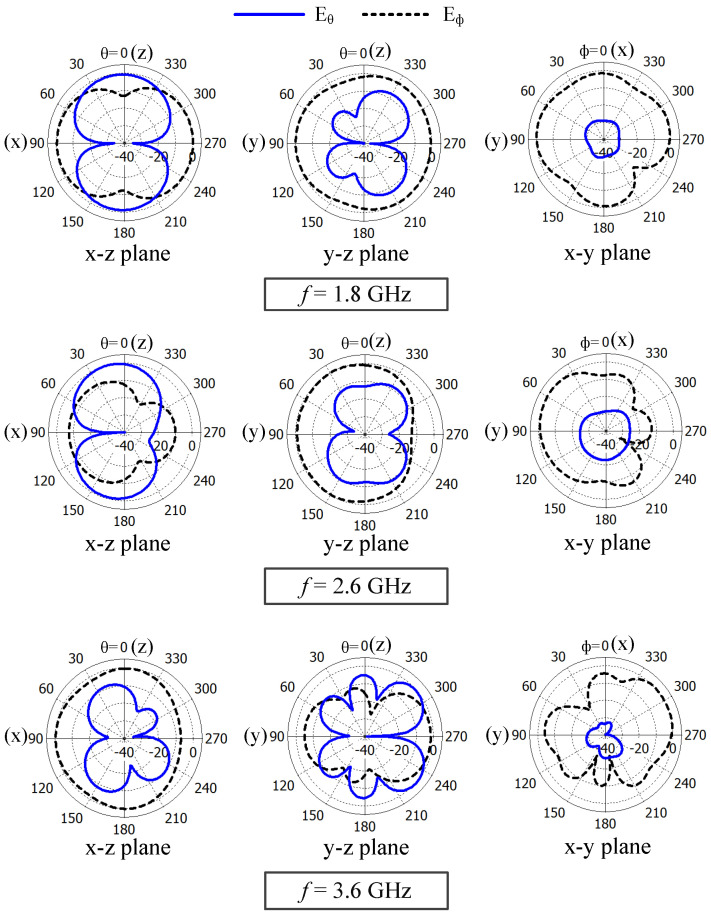
Normalized radiation patterns of the dual-band antenna (Ant#2) at 1.8, 2.6, and 3.6 GHz.

The normalized $E_{\theta }$ and $E_{\phi }$ radiation patterns in the three principal planes at 1.8, 2.6, and 3.6 GHz are shown in Figure 11. The radiation patterns are normalized with respect to the maximum of the corresponding principal plane. The $E_{\phi }$ pattern in the x–z plane at 3.6 GHz is more uniform (nearly omnidirectional) than the $E_{\phi }$ pattern in the same plane at 1.8 and 2.6 GHz. A uniform $E_{\phi }$ pattern can be observed in the y–z plane at 1.8 GHz. Strong $E_{\phi }$ radiation can be observed along the y-axis at 3.6 GHz compared to the z-axis. In addition, the $E_{\phi }$ pattern is more uniform in the x–y plane at 1.8 GHz than the $E_{\phi }$ pattern at 3.6 GHz. The $E_{\phi }$ pattern in the x–y plane has two nulls at 3.6 GHz and one null at 2.6 GHz. Overall, the $E_{\phi }$ component is always stronger than $E_{\theta }$ in the y–z and x–y planes at 1.8 and 2.6 GHz and in the x–z and x–y planes at 3.6 GHz. In addition, the $E_{\phi }$ and $E_{\theta }$ levels are comparable in the x–z plane at 1.8 and 2.6 GHz and in the y–z plane at 3.6 GHz, an additional benefit of practical applications with complex propagation environments [18,24,26].

## 4. Wideband Antenna for LSA Bands and LTE Mid and High Bands

A wideband antenna (Ant#3) is presented in this section. Unlike the previous dual-band antenna (Ant#2), this is a wideband antenna covering both the mid and high bands. In addition, Ant#3 is smaller than both Ant#2 and Ant#1.

### 4.1. Antenna Configuration

**Figure 12:**
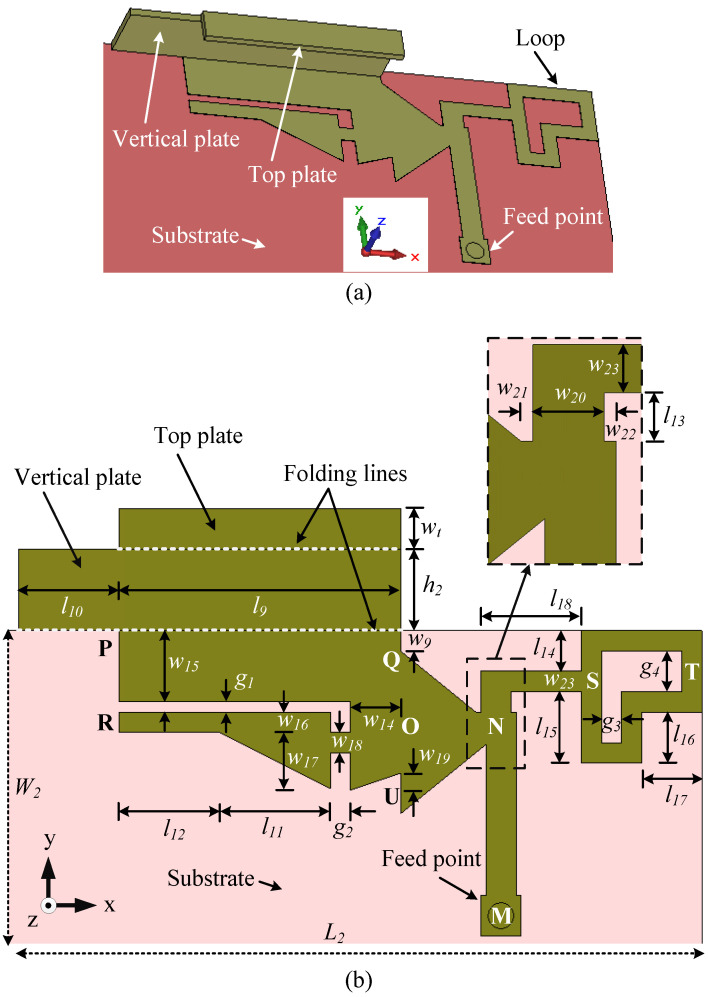
Configuration of the wideband antenna (Ant#3) for the LSA bands, as well as the LTE/WWAN mid and high bands: (**a**) perspective view, and (**b**) top view (vertical plate is unfolded along the +y axis).

Figure 12 shows the configuration of the novel wideband antenna (Ant#3). The overall size of the antenna is 34 mm × 15 mm × 4 mm or 0.16$\lambda_{2}$× 0.07$\lambda_{2}$× 0.02$\lambda_{2}$, where $\lambda_{2}$ is the free-space wavelength at 1.41 GHz. This indicates that the area occupied by the wideband antenna is 15% and 13% smaller than that occupied by Ant#1 and Ant#2, respectively. The substrate size, ground clearance, positioning of the antenna, feed arrangement, and back view are similar to Ant#1, as shown in Figure 1.

Ant#3 is composed of three elements: a U-shaped large element PQOR loaded with an L-shaped vertical plate (aligned with the edge PQ) on the left to cover the mid band, an L-shaped loop element NSTS on the right to cover the high band, and an L-shaped element MNO. It is worth mentioning here that the element MNO is the same as that used in Ant#2. The optimized design parameters of Ant#3 are listed in Table 2. The parameters of the mid-element MNO are presented in Table 1.

### 4.2. Design Procedure and Working Principle

**Figure 13:**
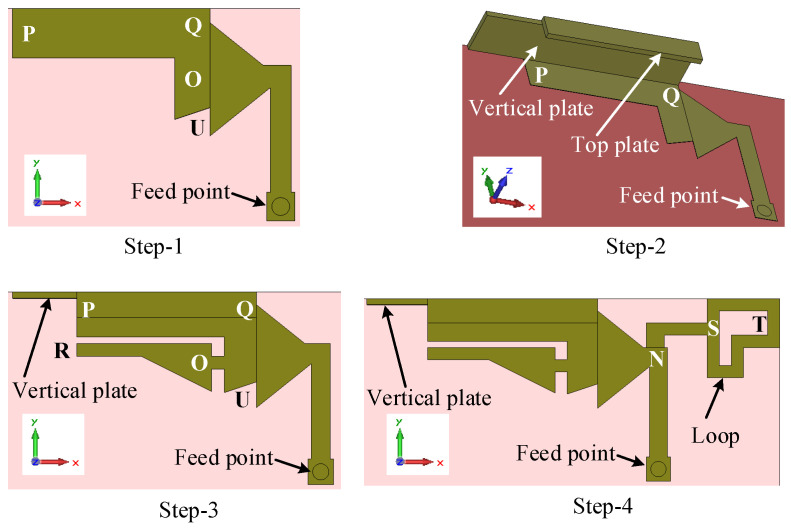
The four steps of the evolution of the wideband antenna (Ant#3).

**Figure 14:**
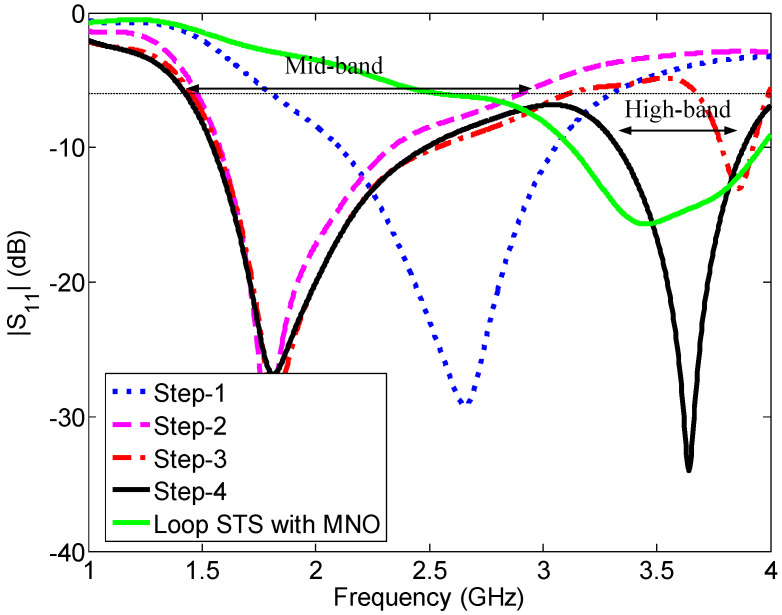
Reflection coefficients for each step in the evolution of the wideband antenna (Ant#3).

In this design approach, our target is to design a more compact antenna than the previous two antennas (Ant#1 and Ant#2) and also remove the nulls from the radiation patterns. The four main steps of the evolution of this antenna are shown in Figure 13. The predicted reflection coefficients for each step are shown in Figure 14.

The working principle of the antenna can be easily described by the design steps. The design of Ant#3 started with a simple planar antenna, where an L-shaped strip UOQP is imposed on the middle element MNO, as shown in Figure 13 (Step 1). This antenna has a −6 dB reflection coefficient bandwidth of 1.58 GHz (1.73–3.31 GHz) and resonated at 2.66 GHz, as shown in Figure 14 (Step 1 curve). Therefore, the antenna is unable to cover the whole LTE mid-band since the lower frequency limit of the LTE mid-band is 1.42 GHz. The effective length of the antenna is $l_{ILE}+w_{14}/2+l_{9}+w_{15}/2+g_{1}+w_{16}=32.7$ mm, which is equal to a quarter-wavelength at 2.66 GHz.

In Step 2, an L-shaped bent plate is loaded onto the antenna for tuning the lower-frequency limit, together with the operating bandwidth. First, an I-shaped vertical plate with a thickness of 0.4 mm is added to the edge PQ, which is aligned with the edge point Q, as shown in Figure 13 (Step-2). The length and height of this vertical plate are $l_{9}+l_{10}$ and $h_{2}$, respectively. Later, a top plate with a size of $l_{9}$ mm × $w_{t}$ mm × 0.4 mm is loaded onto the top of the vertical plate, which is also aligned with the edge point Q, as shown in Figure 13 (Step-2). This antenna resonates at 1.82 GHz and had a −6 dB reflection bandwidth of 1.46 GHz (1.46–2.92 GHz), as shown in Figure 14 (Step-2 curve).

Our next aim is to shift the lower-frequency limit further and generate a second resonant mode in the high band. In Step 3, first, an I-shaped patch OR with a size of $\left(l_{11}+l_{12}\right)\times w_{16}$ is coupled to the L-shaped strip UQP, maintaining a gap of $g_{1}$ and $g_{2}$ along the y- and x-directions, respectively. Second, a triangle-shaped element with a base and height of $l_{11}$ and $w_{17}$, respectively, is added to the bottom-right corner of the element OR. At the end of Step 3, a strip with dimensions of $g_{2}\times w_{18}$ is added to the left of the element UQ. The predicted reflection coefficient (Step 3 curve in Figure 14) shows that a second resonance appears at 3.86 GHz, with a dip in magnitude of −13 dB, and the lower-frequency limit shifts from 1.46 to 1.43 GHz. At the second resonance (3.86 GHz), the antenna resonates in the half-wavelength mode since the effective length of the U-slot antenna POR fed by MNO is $l_{ILA}+w_{14}+2\left(l_{11}+l_{12}+g_{2}\right)=39.7$ mm. The −6 dB reflection bandwidths in the first and second operating bands are 1.67 GHz (1.43–3.1 GHz) and 300 MHz (3.68–3.98 GHz), respectively. The top plate over the element PQ and coupled element OR not only improves the operating bandwidth but also reduces the nulls in the x–y plane radiation patterns at frequencies close to the upper limit of the LTE mid band. Although it improves the x–y plane radiation pattern nulls within the frequency band of 3.7–4 GHz, there is still room for improvement.

Finally, to expand the reflection bandwidth of the antenna at the second resonance in order to cover the high band and to fine-tune the lower-frequency limit of the mid band, an L-shaped loop NSTS with a strip width of $w_{23}$ is integrated into the right side of the middle element in Step 4, as shown in Figure 13. The reflection coefficient curve (Figure 14 (Step 4 curve)) shows that the lower-frequency limit further shifted from 1.43 to 1.41 GHz and the −6 dB reflection coefficient bandwidth of the antenna is 2.59 GHz (1.41–4 GHz) with two resonances at 1.81 and 3.64 GHz. The effective length of the element MNSTS is 41 mm, which is equal to a half-wavelength at 3.65 GHz. The NSTS loop improves the operating bandwidth in the LTE high band and also helps to achieve null-free x–y plane radiation.

Now, let us study the reflection coefficient of the L-shaped loop element NSTS excited by the middle element MNO, as shown in Figure 14 (loop STS with MNO curve), in order to further understand the operation of the right-hand element. This antenna resonates at 3.45 GHz, which is close to the half-wavelength mode since the effective length is 41 mm. In this study, the elements on the left side of QOU (Figure 12b) are removed from the design.

**Figure 15:**
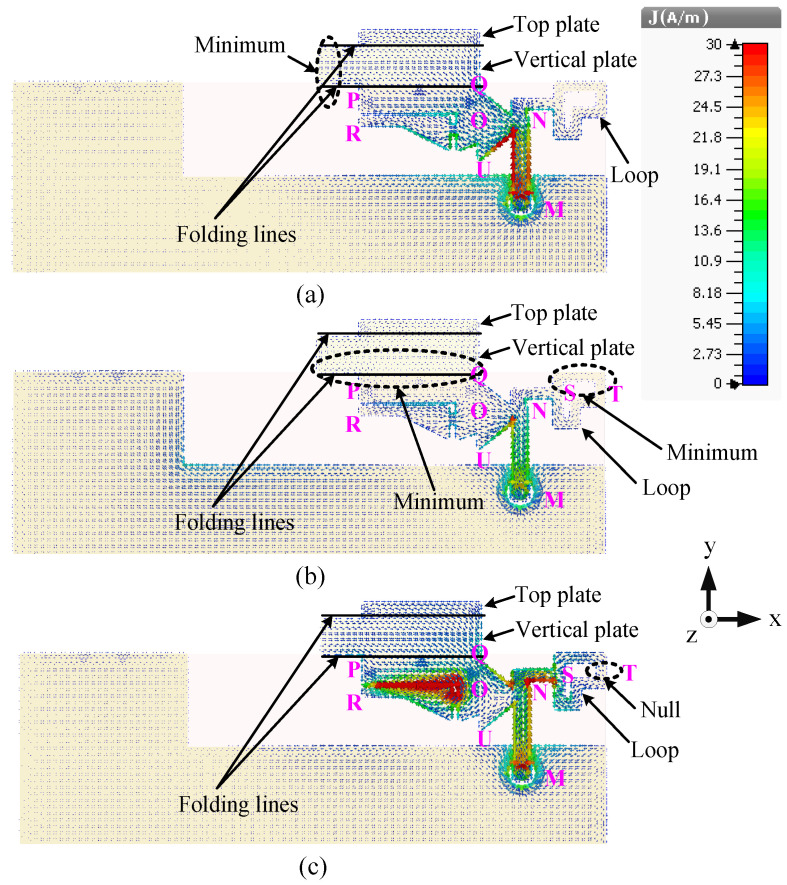
Surface current distributions of the wideband antenna (Ant#3) at (**a**) 1.8, (**b**) 2.6, and (**c**) 3.6 GHz.

The predicted current distributions of this antenna at three frequencies, 1.8, 2.6, and 3.6 GHz, are shown in Figure 15. For better presentation, the vertical L-shaped load is unfolded along the +y axis. It can be seen from Figure 15a that the antenna resonates in the quarter-wavelength mode at 1.8 GHz since only one minimum can be observed (in the vertical plate close to the edge point P). Weak currents can be seen in the loop NSTS at this resonant mode but no nulls is observed. Two minima can be seen (in the vertical plate close to the edge QP and in the upper arms of the loop STS), meaning that the antenna resonates in the half-wavelength mode at 2.6 GHz, as shown in Figure 15b. The current distributions in Figure 15c illustrate that the directions of the currents are the same for the upper and lower arms of the loop STS and a null can be seen at point T. Hence, the loop monopole antenna resonates in the half-wavelength mode at 3.6 GHz. In addition, the left element PQUR has a strong current at 3.6 GHz but no null can be observed.

## 5. Measured and Predicted Results

**Figure 16:**
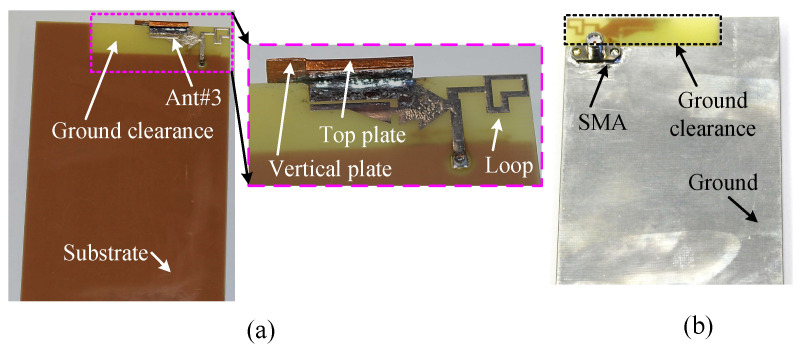
Fabricated wideband antenna (Ant#3) for mid and high bands: (**a**) top view of the antenna with whole mobile device substrate (antenna is shown in the inset), and (**b**) bottom view (whole substrate).

This section presents the measured reflection coefficient, efficiency, gain, and radiation patterns of the wideband antenna (Ant#3), which are compared with the predicted results. The radiation performance of the fabricated antenna was measured using the NSI700S-50 near-field range at the Australian Antenna Measurement Facility, CSIRO, Marsfield, N.S.W., and the reflection coefficients were measured using an Agilent Network Analyzer PNA-X N5242A at Macquarie University, Australia. The top and bottom views of the prototype are shown in Figure 16.

### 5.1. Reflection Coefficient

**Figure 17:**
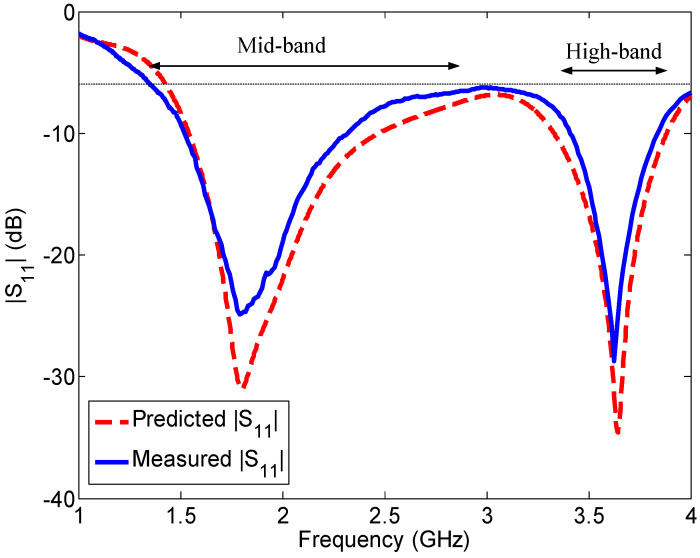
Measured and predicted reflection coefficients of the wideband antenna (Ant#3).

The measured reflection coefficient of Ant#3 is shown in Figure 17, together with the predicted results. Excellent agreement is observed between the measured and the predicted results. The −6 dB (VSWR < 3) reflection coefficient bandwidth of the prototyped antenna (Ant#3) is 2.63 GHz (1.37–4 GHz), which covers the two LSA, 16 FDD LTE, 11 TDD LTE, UMTS, DCS/GSM1800, PCS/GSM1900, 2.4 GHz WLAN, and 3.3 GHz WiMAX frequency bands.

### 5.2. Radiation Performance

**Figure 18:**
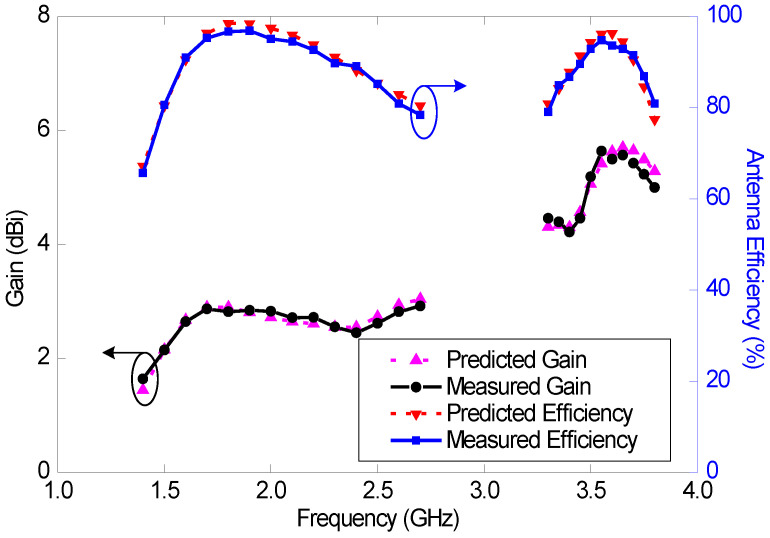
Measured and predicted gains and efficiencies of the wideband antenna (Ant#3).

The measured efficiency and gain, together with the predicted results of the wideband antenna (Ant#3), are shown in Figure 18. A gain comparison method was used for the antenna gain measurements. Again, a good agreement between the measured and predicted results is observed. The measured maximum efficiencies of this antenna in the mid and high bands are 96% and 94%, respectively, and the minimum efficiencies are 66% and 79%, respectively. The measured maximum gains within the reflection coefficient bandwidth (VSWR < 3) of this antenna in the mid and high bands are 2.9 and 5.6 dBi, respectively, and the minimum gains are 1.6 and 4.2 dBi, respectively.

**Figure 19:**
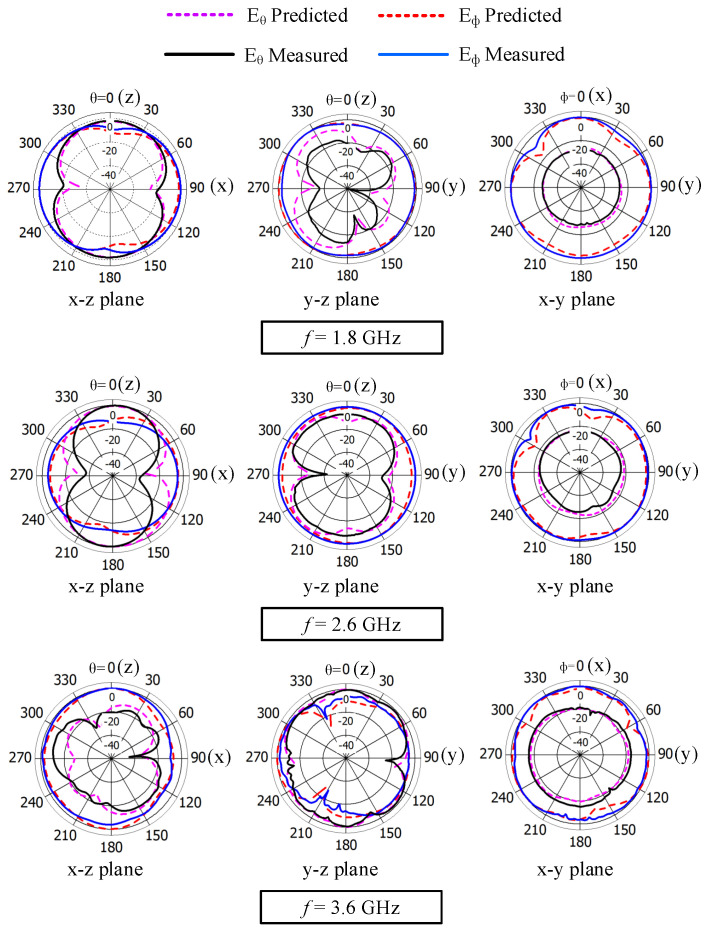
Measured and predicted normalized radiation patterns of the wideband antenna (Ant#3) at 1.8, 2.6, and 3.6 GHz.

The measured $E_{\theta }$ and $E_{\phi }$ radiation patterns in the three principal planes at 1.8, 2.6, and 3.6 GHz are shown in Figure 19 and compared with the predicted results. The radiation patterns are normalized with the maximum value of the corresponding plane. A good agreement between the predicted and measured results is found. At 1.8 and 2.6 GHz, strong $E_{\phi }$ radiation is observed in the x–y and y–z planes compared to $E_{\theta }$ radiation, and at 3.6 GHz, strong $E_{\phi }$ radiation is observed in the x–z and x–y planes compared to $E_{\theta }$ radiation. The radiation is nearly omnidirectional in the y–z and x–y planes in the mid band and in the x–z and z–y planes in the high band. In addition, there are no nulls in the radiation patterns of the mid and high bands. In addition, the $E_{\theta }$ and $E_{\phi }$ levels are comparable in both the x–z plane at the mid-band frequencies and the y–z plane at the high-band frequencies, which makes the antenna suitable for practical applications with complex propagation environments [18,24,26].

### 5.3. Comparison of Antennas

The properties of the dual-band antenna (Ant#2) and the wideband antenna (Ant#3) proposed in this paper are summarized, together with the LSA antenna (Ant#1), in Table 3. The efficiency of Ant#3 is higher than that of Ant#2 in both the mid and high bands. The gain variations of Ant#3 are 1.3 and 1.4 dB in the mid and high bands, respectively, whereas the variations are 2 and 1.9 dB, respectively, for Ant#2. The radiation patterns in the mid band are nearly omnidirectional for both antennas (Ant#2 and Ant#3). However, in the high band, Ant#2 had nearly omnidirectional radiation patterns with two nulls in the x–y plane radiation patterns. On the other hand, Ant#3 had a nearly omnidirectional pattern in the high band without any nulls.

A comparison of the fabricated antenna (Ant#3) and the reference antennas is provided in Table 4 in terms of performance and the area occupied on the substrate to verify the suitability of the proposed antennas for hand-held devices. The comparison in Table 4 shows that Ant#3 has high efficiency and a large bandwidth than most of the reference antennas to cover the mid and high bands while occupying a smaller area.

## 6. Conclusions

First, a planar antenna was designed for application in mobile devices. The operating bandwidth of the planar antenna is 1170 MHz (1.38–2.55 GHz), which covers both LSA frequency bands. Then, two novel antennas were designed to cover both LSA bands (1452–1492 and 2300–2400 MHz) and the mid- (1427–2690 MHz) and high-LTE (3300–3800 MHz) bands. Although these antennas have a small vertical plate at the edge of the radiating element, the areas occupied by the dual-band and wideband antennas are 3% and 15% smaller than most of the reference planar antennas. There are nulls in the radiation patterns of the planar antenna and the dual-band antenna. The radiation patterns of the wideband antenna do not have any nulls. Finally, in order to prove the concept, the wideband antenna was fabricated and measured. The measured −6 dB impedance bandwidth of the prototype is 1.37–4 GHz, which covers all the targeted frequency bands. 

## Figures and Tables

**Table 1:** Design parameters of the dual-band antenna (Ant#2). All dimensions are in millimeters.

$w_{1}=2$	$w_{2}=1.5$	$w_{3}=1.6$	$w_{4}=0.5$	$w_{5}=3$
$w_{6}=3.4$	$w_{7}=2$	$w_{8}=1$	$w_{9}=1$	$w_{10}=3$
$w_{11}=4$	$w_{12}=1.5$	$l_{1}=7.4$	$l_{2}=4.25$	$l_{3}=22$
$l_{4}=11$	$l_{5}=7.75$	$l_{6}=4$	$h_{1}=4$	r=2
$L_{1}=39$	$W_{1}=15$			

**Table 2:** Design parameters of the wideband antenna (Ant#3). All dimensions are in millimeters.

$w_{9}=1$	$w_{14}=2.5$	$w_{15}=3.5$	$w_{16}=1$	$w_{17}=2.75$
$w_{18}=1$	$w_{19}=0.75$	$w_{20}=1.5$	$w_{21}=0.25$	$w_{22}=0.25$
$w_{23}=1$	$l_{9}=14$	$l_{10}=5$	$l_{11}=5.5$	$l_{12}=5$
$l_{13}=1$	$l_{14}=2$	$l_{15}=3.5$	$l_{16}=2.5$	$l_{17}=3$
$l_{18}=5$	$g_{1}=0.5$	$g_{2}=1$	$g_{3}=1$	$g_{4}=2$
$h_{2}=4$	$w_{t}=2$	r=2	$L_{2}=34$	$W_{2}=15$

**Table 3:** Performance comparison of the presented LSA (Ant#1), dual-band (Ant#2), and wideband (Ant#3) antennas.

Parameters	LSA Antenna	Dual-Band Antenna	Wideband Antenna
	(Ant#1)	(Ant#2)	(Ant#3)
Physical Area (mm${}^{2}$)	600	585	510
Bandwidth (GHz)	1.38–2.55	1.39–2.84, 3.27–3.97	1.37–4
Efficiency (%)	66–89	62–90 (M), 66–88 (H) ${}^{*}$	66–96 (M), 79–94 (H)
Gain (dBi)	1.3–3.6	1.4–3.4 (M), 4.2–6.1 (H)	1.6–2.9 (M), 4.2–5.6 (H)
Nulls in Rad. Pat.	In x–y plane	In x–y plane (H)	-

* M and H stand for the LTE mid and high bands, respectively.

**Table 4:** Performance comparison of the reference antennas and the proposed wideband antenna for the LTE mid and high bands.

Ref.	Efficiency * (%)	Bandwidth (GHz)	Antenna Height	Area	Substrate (Thickness)
[16]	64–83 (M)	1.68–2.74	5	3360	FR4 (0.8 mm)
[17]	50–73 (M)	1.63–2.74	5.8	2240	FR4 (0.8 mm)
[18]	65–83 (M)	1.71–2.69	7	567	FR4 (0.4 mm)
[22]	50–93 (M), 53–73 (H)	1.69–4.02	7	800	FR4 (0.8 mm)
[23]	48–84 (M), 62–91 (H)	1.71–2.69, 3.4–3.8	6	2400	FR4 (0.8 mm)
[24]	60–75 (M), 78–91 (H)	1.71–3.85	5	560	FR4 (0.8 mm)
[25]	59–90 (M), 50–85 (H)	1.71–3.02, 3.37–3.9	5	1125	FR4 (1.6 mm)
[27]	>50 (M and H)	1.7–2.9, 3.4–3.8	5	2812	FR4 (0.8 mm)
[31]	58–77 (M)	1.66–2.77	3	550	FR4 (1 mm)
[32]	57–87 (M)	1.6–2.7	4	525	FR4 (1 mm)
[33]	Not given	1.48–2.176, 2.28–2.775,	0.8	1020	FR4 (0.8 mm)
		2.97–4.98, 5.125–6			
[34]	40–66 (M)	1.71–2.85	0.8	874	FR4 (0.8 mm)
[35]	88–95 (M) (rad)	1.67–2.87	6	560	FR4 (0.8 mm)
Ant#3	66–96 (M), 79–94 (H)	1.37–4	4	510	FR4 (0.8 mm)

* M and H stand for the mid and high bands, respectively. Area = length × width (mm^2^). The antenna height is in millimeters.

## Data Availability

Not applicable.
